# *SLC25A11*, a Novel Gene Associated With Carney-Stratakis Syndrome

**DOI:** 10.1210/jendso/bvaf052

**Published:** 2025-04-02

**Authors:** Felipe Freitas-Castro, Lucas S Santana, Gustavo F C Fagundes, Eduardo C Lobato, Ana Caroline F Afonso, Izabel T Nakamura, Felipe L Ledesma, Ibere C Soares, Berenice B Mendonca, Ana Claudia Latronico, Constantine A Stratakis, Madson Q Almeida

**Affiliations:** Unidade de Adrenal, Laboratório de Endocrinologia Molecular e Celular LIM25, Divisão de Endocrinologia e Metabologia, Hospital das Clínicas HCFMUSP, Faculdade de Medicina da Universidade de São Paulo, São Paulo 01246-903, Brasil; Unidade de Adrenal, Laboratório de Endocrinologia Molecular e Celular LIM25, Divisão de Endocrinologia e Metabologia, Hospital das Clínicas HCFMUSP, Faculdade de Medicina da Universidade de São Paulo, São Paulo 01246-903, Brasil; Unidade de Adrenal, Laboratório de Endocrinologia Molecular e Celular LIM25, Divisão de Endocrinologia e Metabologia, Hospital das Clínicas HCFMUSP, Faculdade de Medicina da Universidade de São Paulo, São Paulo 01246-903, Brasil; Unidade de Adrenal, Laboratório de Endocrinologia Molecular e Celular LIM25, Divisão de Endocrinologia e Metabologia, Hospital das Clínicas HCFMUSP, Faculdade de Medicina da Universidade de São Paulo, São Paulo 01246-903, Brasil; Laboratório de Hormônios e Genética Molecular LIM42 e Laboratório de Sequenciamento em Larga Escala (SELA), Divisão de Endocrinologia e Metabologia, Hospital das Clínicas HCFMUSP, Faculdade de Medicina da Universidade de São Paulo, São Paulo 01246-903, Brasil; Unidade de Adrenal, Laboratório de Endocrinologia Molecular e Celular LIM25, Divisão de Endocrinologia e Metabologia, Hospital das Clínicas HCFMUSP, Faculdade de Medicina da Universidade de São Paulo, São Paulo 01246-903, Brasil; Divisão de Anatomia Patológica, Hospital das Clínicas HCFMUSP & Instituto do Câncer do Estado de São Paulo (ICESP), Faculdade de Medicina da Universidade de São Paulo, São Paulo 01246-903, Brasil; Divisão de Anatomia Patológica, Hospital das Clínicas HCFMUSP & Instituto do Câncer do Estado de São Paulo (ICESP), Faculdade de Medicina da Universidade de São Paulo, São Paulo 01246-903, Brasil; Laboratório de Hormônios e Genética Molecular LIM42 e Laboratório de Sequenciamento em Larga Escala (SELA), Divisão de Endocrinologia e Metabologia, Hospital das Clínicas HCFMUSP, Faculdade de Medicina da Universidade de São Paulo, São Paulo 01246-903, Brasil; Unidade de Adrenal, Laboratório de Endocrinologia Molecular e Celular LIM25, Divisão de Endocrinologia e Metabologia, Hospital das Clínicas HCFMUSP, Faculdade de Medicina da Universidade de São Paulo, São Paulo 01246-903, Brasil; Human Genetics & Precision Medicine, IMBB, FORTH, Heraklion, Crete & ASTREA Health, Athens 11528, Greece; Unidade de Adrenal, Laboratório de Endocrinologia Molecular e Celular LIM25, Divisão de Endocrinologia e Metabologia, Hospital das Clínicas HCFMUSP, Faculdade de Medicina da Universidade de São Paulo, São Paulo 01246-903, Brasil; Unidade de Oncologia Endócrina, Instituto do Câncer do Estado de São Paulo (ICESP), Faculdade de Medicina da Universidade de São Paulo, São Paulo 01246-000, Brasil

**Keywords:** pheochromocytoma, paraganglioma, genetics, Carney-Stratakis syndrome

## Abstract

**Background:**

Carney-Stratakis syndrome (CSS), a rare condition characterized by paragangliomas and/or pheochromocytomas and gastrointestinal stromal tumors (GIST), is caused by germline heterozygous pathogenic variants in the succinate dehydrogenase subunit genes (*SDHB, SDHC, SDHD*).

**Methods:**

Histological, genetic, and functional analyses were conducted in a 59-year-old female with CSS (9 cm left pheochromocytoma, 4.8 cm paraganglioma, and 9.3 cm GIST). Whole-exome sequencing (WES) of germline DNA paired with tumor DNA was performed.

**Results:**

WES identified a rare heterozygous germline variant (c.293G>A/p.Arg98His) in the mitochondrial 2-oxoglutarate/malate carrier gene (*SLC25A11*). This variant, located in a highly conserved residue of the *SLC25A11* mitochondrial carrier domain, is predicted to be deleterious in silico (REVEL score = 0.81). WES of pheochromocytoma, paraganglioma, and GIST did not reveal somatic pathogenic variants in genes previously associated with these tumors. A significant reduction in *SLC25A11* expression was observed in the tumors of this patient with the *SLC25A11* c.293G>A variant (0.69 ± 0.003) compared to tumors from cluster 1 (1.39 ± 0.45; *P* = 0.0229) and cluster 2 (1.79 ± 0.71; *P* = .0154). Consistent with the mRNA findings, *SLC25A11* protein levels were markedly reduced in the pheochromocytoma and paraganglioma compared to other tumors. Negative staining for 5-hydroxymethylcytosine in all 3 tumors suggests a DNA hypermethylation profile characteristic of cluster 1A, despite normal SDHB expression levels. However, genome-wide copy number variation analysis did not reveal any loss of heterozygosity at the *SLC25A11* locus.

**Conclusion:**

The loss of SLC25A11 expression in tumors, the absence of somatic drivers, and the hypermethylation status strongly support the role of *SLC25A11* in CSS pathogenesis.

Carney-Stratakis syndrome (CSS, OMIM #606864), also known as the Carney-Stratakis dyad, is characterized by the co-occurrence of pheochromocytomas and/or paragangliomas alongside gastrointestinal stromal tumors (GISTs). This syndrome was first described in 2002 by Carney and Stratakis and can affect both male and female individuals during childhood and adolescence [1]. CSS is caused by germline heterozygous loss-of-function pathogenic variants in the succinate dehydrogenase subunit genes, specifically *SDHB, SDHC*, and *SDHD*, with *SDHB* and *SDHD* being the most commonly affected [2-5].

The Carney triad (OMIM #604287) is defined by the association of paraganglioma, GIST, and pulmonary chondroma. Unlike Carney-Stratakis syndrome, it is not caused by germline pathogenic variants in the *SDHx* genes [6]. Instead, *SDHC*-specific hypermethylation in the promoter and first exon is considered the molecular signature of GISTs associated with the Carney triad and wild-type GISTs [7, 8]. Very rare cases of CSS patients without genetic defects in *SDHB*, *SDHD*, or *SDHC* have previously been described [2, 9]. However, these cases were not thoroughly investigated using multiplex ligation-dependent probe amplification (MLPA) or next-generation sequencing to rule out large deletions and to identify potential new susceptibility genes for CSS. Recently, a germline *SDHC* exon 3 deletion was identified in a patient with CCS [10].

In this study, we investigated a woman who presented with pheochromocytoma, paraganglioma, and GIST, fulfilling the criteria for CSS but lacking pathogenic variants or gene deletions in the *SDHB, SDHD,* or *SDHC* genes. Whole-exome sequencing (WES) of paired blood and tumor samples, conducted to explore the genetic etiology of CSS, revealed a novel heterozygous germline variant (c.293G>A/p.R98H) in the solute carrier family 25 member 11 (*SLC25A11*) gene, which encodes the mitochondrial 2-oxoglutarate/malate carrier protein. We conducted detailed histological, genetic, and functional analyses supporting the involvement of *SLC25A11* in the pathogenesis of CSS.

## Methods

This study was approved by the Ethics Committee of the Clinics Hospital (#06194919.1.0000.0068) and the Cancer Institute of São Paulo State (#1448/19), University of São Paulo Medical School. A written informed consent form was signed by the patient. The biochemical and imaging diagnoses of pheochromocytoma and/or paraganglioma (PPGL) followed the Endocrine Society's recommendations [11]. Metastatic PPGL was defined as the presence of tumors in nonchromaffin tissues. The TNM staging system for PPGLs developed by the American Joint Committee on Cancer was used [12, 13].

### Case Report

**Figure 1.**
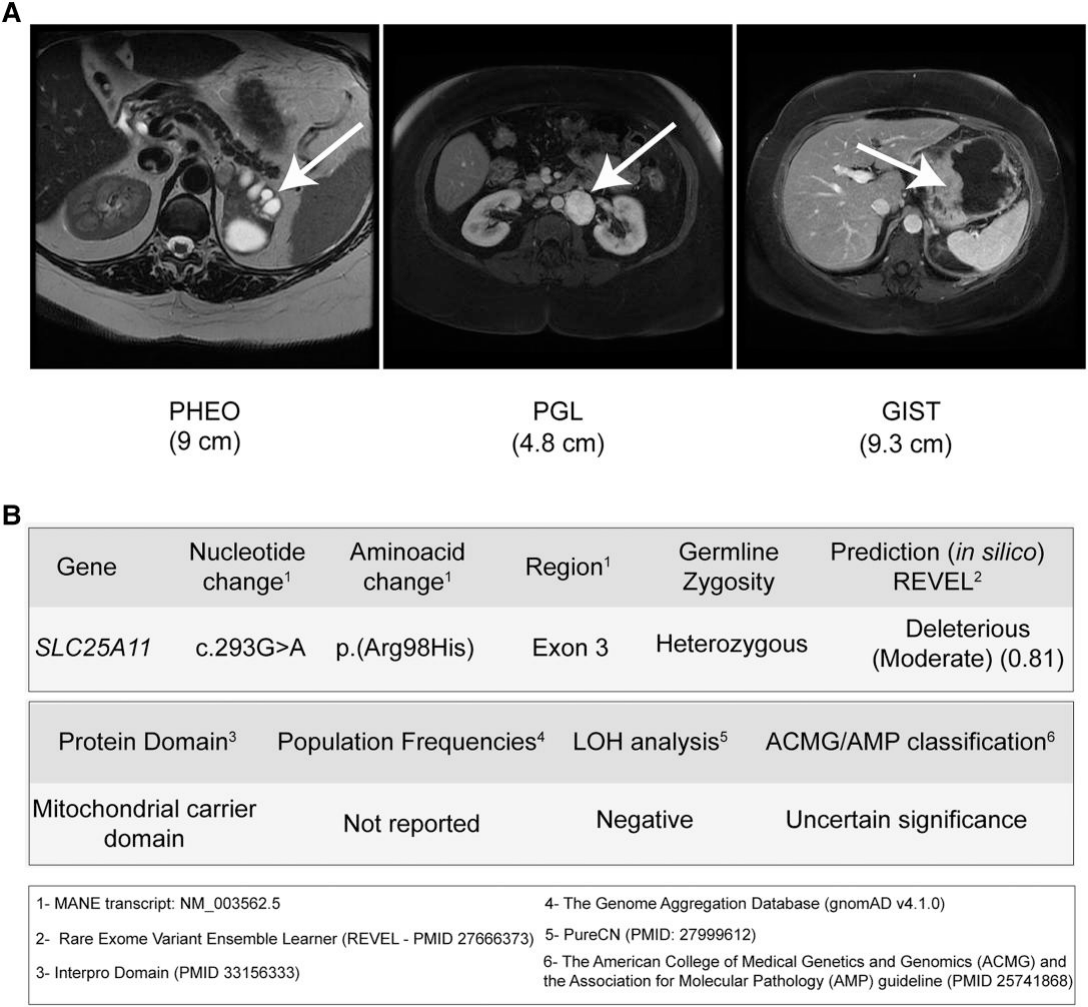
(A) Magnetic resonance images of a patient with Carney-Stratakis syndrome. Axial T2-weighted magnetic resonance imaging (MRI) shows a left solid-cystic pheochromocytoma (PHEO; white arrow), an axial T2-weighted MRI depicts a para-aortic paraganglioma (PGL; white arrow), and MRI scans illustrate a gastrointestinal stromal tumor (GIST; white arrow) originating from the gastric body and fundus. (B) Whole-exome sequencing results reveal a key germline variant in the DNA of the patient with CSS.

A 59-year-old woman was incidentally found to have 2 lesions—a retroperitoneal mass and a left adrenal tumor—during a routine ultrasound. She did not exhibit symptoms such as adrenergic paroxysms, headache, abdominal pain, or intestinal obstruction. The patient had a history of severe obesity, stage 2 arterial hypertension, and type 2 diabetes mellitus, which were diagnosed at age 35. Additionally, she underwent cholecystectomy and partial gastrectomy due to a gastric ulcer 20 years prior. There was no family history of GIST, pheochromocytoma, or paraganglioma. Abdominal magnetic resonance imaging (MRI) revealed a left hypervascular para-aortic retroperitoneal mass measuring 4.8 cm, as well as a solid-cystic lesion measuring 9.0 cm in the left adrenal gland. Additionally, the MRI showed a 9.3 cm mass originating from the gastric body and fundus, suggestive of mesenchymal neoplasia (GIST) (Fig. 1A). Plasma metanephrine levels were measured at <0.2 nmol/L (38.46 pg/mL), within the normal range of <0.5 nmol/L (96.15 pg/mL), while normetanephrine levels were elevated at 1.5 nmol/L (288.45 pg/mL), exceeding the normal range of <0.9 nmol/L (173.07 pg/mL). A meta-iodine-benzyl-guanidine scan labeled with iodine 131 (^131^I-MIBG) demonstrated high uptake in the left para-aortic and adrenal lesions. A chest computed tomography did not reveal any pulmonary chondromas, and other imaging excluded other tumors, effectively ruling out a diagnosis of Carney triad.

After 3 weeks of α-adrenergic blockade, she underwent successful subtotal gastrectomy, left adrenalectomy, and resection of the para-aortic lesion. Histopathological analysis identified pheochromocytoma (PASS score = 4, Ki-67 index <1%, positive for chromogranin and synaptophysin), paraganglioma (Ki-67 index 1%-2%, also positive for chromogranin and synaptophysin), and an epithelioid GIST (mitotic count: > 3/5 mm^2^) that tested positive for KIT (CD117), CD34, and DOG1. Immunohistochemical staining for SDHB was positive in all 3 tumors [10]. Six peritoneal lymph nodes were also resected. A metastatic abdominal lymph node was detected with positive staining for chromogranin and synaptophysin.

Following surgery, plasma normetanephrine levels returned to normal, and MIBG uptake was negative. After 2 years of follow-up, there was no recurrence of paraganglioma or pheochromocytoma; however, she did show progression of peritoneal lymph node metastases from the GIST, confirmed by biopsy. Initial genetic testing using a targeted next-generation sequencing panel and MLPA did not identify any pathogenic variants in the *SDHB, SDHD, SDHC, SDHA,* and *SDHAF2* genes [10].

### DNA, RNA, and Protein Extraction

Germline DNA was extracted from the peripheral blood using the salting-out method. Tumor tissues, obtained during routine surgical procedures, were stored in liquid nitrogen until genetic material was extracted. DNA, RNA, and protein were extracted using AllPrep DNA/RNA/Protein Mini Kit (Qiagen).

### Whole-Exome Sequencing

Whole-exome sequencing (WES) was conducted using a DNA nanoball sequencing assay (DNBSEQ, BGI Genomics Europe, Herlev, Denmark) after somatic and germline exome enrichment with KAPA HyperExome Probes (Roche, CA, USA), ensuring high-quality sequencing with low duplicate rate and deep coverage. Bioinformatic analysis was conducted as previously described. WES and targeted panel sequencing data were screened for rare variants with minor allele frequency of less than 0.1% in population public databases, including the Genome Aggregation Database (global) and the Brazilian genomic variant repository (ABraOM) (local). Additionally, rare nonsynonymous coding and consensus splice-site variants were selected using a targeted approach, focusing on 3485 potential neuroendocrine tumor susceptibility genes, associated with pathways such as cellular response to hypoxia, mitochondrion, Krebs cycle, and tumorigenesis (MAPK, mTOR, ERK, and Wnt signaling). Germline variants were classified according to the American College of Medical Genetics and Genomics (ACMG) and the Association for Molecular Pathology (AMP) guideline [14].

### Real-Time Quantitative Polymerase Chain Reaction

For real-time quantitative polymerase chain reaction (RT-qPCR), RNA was extracted from tumor tissues using 2 different kits: the AllPrep DNA/RNA Mini Kit and the AllPrep DNA/RNA/Protein Mini Kit (both from Qiagen). Extracted RNA was treated with ezDNase (Thermo Fisher Scientific) to eliminate any residual genomic DNA contamination. Complementary DNA (cDNA) synthesis was performed using SuperScript IV reverse transcriptase (Thermo Fisher Scientific) with oligo (dT) primers, following the manufacturer's instructions.

For relative quantification, TaqMan Fast Advanced Master Mix (Thermo Fisher Scientific) was used in conjunction with commercially available TaqMan assays (Thermo Fisher Scientific) for the following target genes: *SLC25A11* (HS01087948_g1), *SDHB* (HS00268117_m1), and the endogenous control *ACTB* (HS03023943_g1). Amplification and detection were carried out on a StepOnePlus Real-Time PCR system (Thermo Fisher Scientific). Data analysis was performed using the ΔΔCt method. ACTB was used as the endogenous control for normalization.

### Protein Extraction and Western Blot Assay

Proteins from tumor tissue samples were extracted using the AllPrep DNA/RNA/Protein Mini Kit (Qiagen). Protein concentrations were determined using the BCA colorimetric assay (Thermo Fisher Scientific). A total of 30 μg of protein from each sample was resolved on a 12% SDS-PAGE gel and transferred onto Amersham Protran nitrocellulose membranes (10600002, Cytiva) using the Mini-PROTEAN Tetra System transfer cell (#1658006, BioRad). Membranes were blocked with TBS-T containing 5% skimmed milk for 2 to 16 hours, followed by incubation with primary antibodies for 2 to 16 hours at concentrations specified by the manufacturers.

The primary antibodies used were as follows: anti-SDHB (21A11AE7, Thermo Fisher Scientific; RRID: AB_2532233), anti-β-Tubulin (D10, sc-5274, Santa Cruz Biotechnology; RRID: AB_2288090), anti-β-Actin (C4, sc-47778, Santa Cruz Biotechnology; RRID: AB_626632), and anti-SLC25A11 (12253-1-AP, Proteintech, RRID: AB_2877840). Secondary antibodies conjugated to horseradish peroxidase (HRP) were Amersham ECL anti-rabbit (NA934, Cytiva; RRID: AB_772206) and Amersham ECL anti-mouse (NA931, Cytiva; RRID: AB_772210), both diluted 1:10 000 and incubated for 1 hour. The blots were developed using the ECL Prime Western Blotting Detection System (GERPN2232, Cytiva), and the images were captured using the ChemiDoc Imaging System (#12003153, BioRad).

### Immunohistochemistry

Immunohistochemical analysis was conducted on formalin-fixed, paraffin-embedded (FFPE) tumor tissues to evaluate SDHB protein expression and the epigenomic DNA demethylation marker 5-hydroxymethylcytosine (5hmC) in 3 tumors from a patient diagnosed with CSS. The FFPE tissue sections were deparaffinized, rehydrated, and subjected to antigen retrieval in citrate buffer (pH 6.0) at 95 °C for 20 minutes. Endogenous peroxidase activity was quenched using 3% hydrogen peroxide, followed by blocking of nonspecific binding with 5% bovine serum albumin (BSA). The sections were incubated overnight at 4 °C with primary antibodies targeting SDHB (HPA002868, Sigma-Aldrich; RRID: AB_1079889), and 5hmC (MA5-24695, Thermo Fisher Scientific; RRID: AB_2665308). Protein expression was evaluated using a semi-quantitative scoring system: negative, weak (≤25% positive cells), moderate (25%-50% positive cells), and strong (≥50% positive cells).

### Genome-Wide Copy Number Variation Analysis

Genome-wide copy number variation (CNV) analysis was performed on selected tumor samples (frozen) using the Infinium CytoSNP-850K v1.1 BeadChip microarray (Illumina), which interrogates approximately 850 000 single-nucleotide polymorphisms (SNPs). Genomic DNA was subjected to whole-genome amplification and hybridized onto the BeadChip, following the manufacturer's protocol. Arrays were scanned using the NextSeq 550 system (Illumina, San Diego, CA, USA).

Genotyping data were visualized, normalized, and clustered using the Genotyping Module of the GenomeStudio software v.2.0.5 (Illumina, San Diego, CA, USA) with CytoSNP-850K v1.4 Support Files, as previously described [15]. CNV detection was conducted using the cnvPartition v3.2.1 algorithm (Illumina, San Diego, CA, USA), which calculates the log R ratio (LRR), reflecting the observed-to-expected probe intensity ratio, and the B-allele frequency (BAF). Deviations in LRR and BAF values were indicative of potential copy number alterations. Chromosome positions were mapped according to the human genome assembly GRCh37 (NCBI)/hg19 (UCSC). Additionally, the PureCN algorithm was also utilized for the CNV analysis in the tumor samples studied, as previously reported [15].

### Statistical Analysis

Descriptive statistics were reported as absolute counts (n) and relative frequencies (%) for qualitative variables and as medians with interquartile ranges for quantitative variables. Quantitative variables were compared between 2 groups using a *t* test. All hypotheses were 2-sided and tested at a 5% significance level, with a *P* value <.05 considered statistically significant. Calculations were performed using SPSS software (Version 25.0; SPSS Inc., Chicago, IL, USA).

## Results

### Molecular Diagnosis in the Clinical Case With CSS

The molecular pathogenesis of a patient with CSS was investigated using a range of molecular diagnostic techniques. First, no pathogenic, likely pathogenic, or variants of uncertain significance were detected in the *SDHB, SDHC*, or *SDHD* genes in the patient's germline DNA using Sanger sequencing [10]. No structural variants, such as exon deletions or amplifications, were detected in the *SDHB*, *SDHC*, *SDHD*, and *SDHAF2* genes by multiplex ligation-dependent probe amplification (MLPA) analysis (Supplementary Fig. S1) [16].

Germline DNA and somatic DNA from tumor tissues (pheochromocytoma, paraganglioma, and GIST) were subjected to WES. The WES revealed a novel germline heterozygous variant of uncertain significance (c.293G>A/p.R98H) in *SLC25A11* (Fig. 1B). This variant occurs at a highly conserved residue within the mitochondrial transporter domain and is predicted to be deleterious based on in silico analysis (REVEL score = 0.81). Notably, this variant is absent from population genomic databases (Fig. 1B). Furthermore, an active search revealed no somatic pathogenic variants in genes previously implicated in PPGL tumors, including *SDH*x genes associated with CSS. WES identified only 2 somatic *TP53* pathogenic variants in the GIST, potentially contributing to its aggressive behavior. Additionally, no somatic mutations were found in *KIT* or *PDGFRA* genes in the GIST sample.

### *SDHB* Expression in Tumor Samples

Despite the absence of pathogenic variants in the *SDH*x genes, we aimed to further confirm *SDHB* expression in the pheochromocytoma and paraganglioma from the patient using RT-qPCR and Western blot analysis. Protein and mRNA samples were not available from the GIST. These analyses were performed to definitively exclude *SDHB* deficiency as a contributing factor in the pathogenesis of pheochromocytoma and paraganglioma in this patient with CSS.

**Figure 2.**
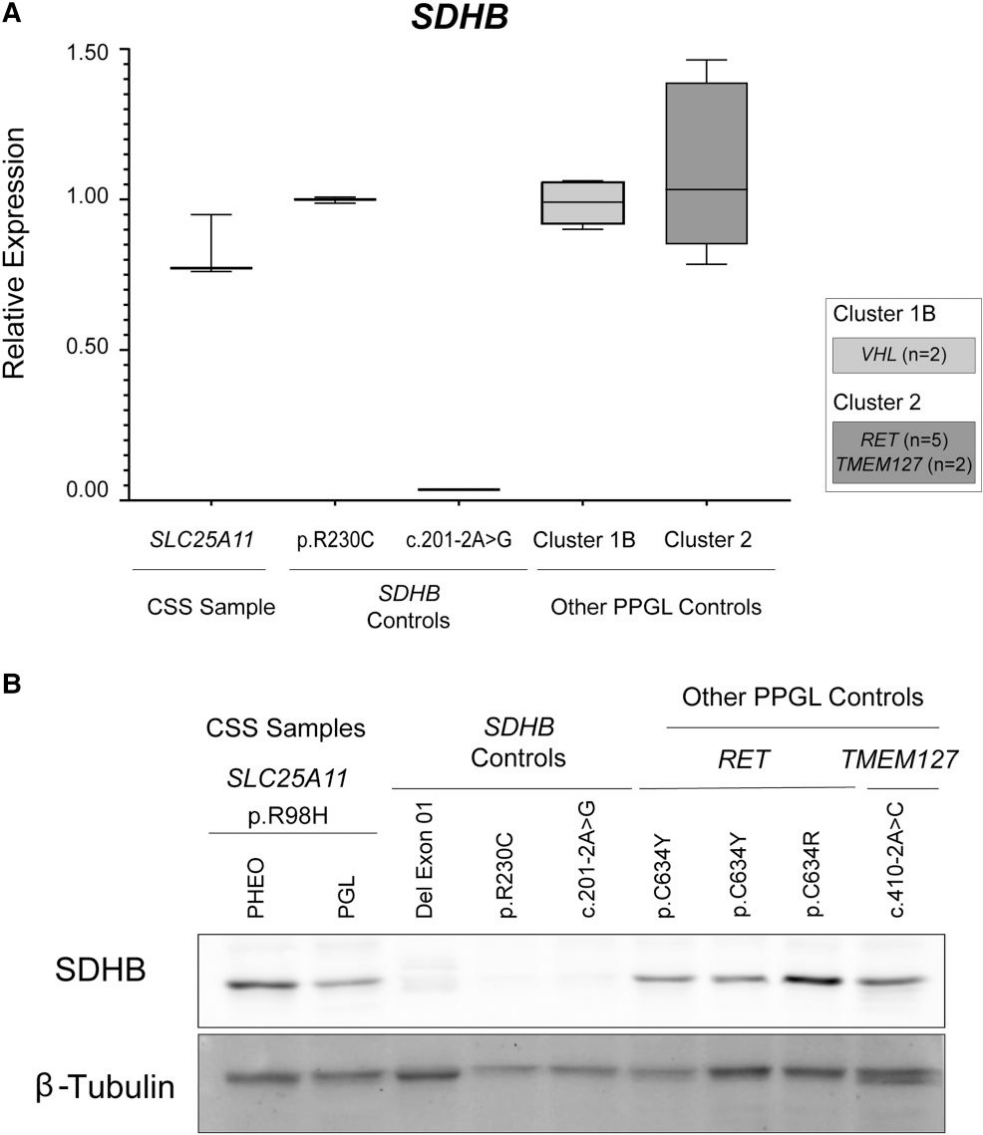
(A) Analysis of *SDHB* transcript levels in PPGL tumor samples using RT-qPCR. Bars represent the mean ± SD between replicates. CSS sample (patient sample): Refers to the RNA obtained from the paraganglioma (PGL) and pheochromocytoma (PHEO) of a patient with Carney-Stratakis syndrome harboring the novel heterozygous germline variant c.293G>A (p.R98H) in *SLC25A11*. SDHB Controls: Includes tumor samples from patients carrying pathogenic *SDHB* variants, specifically p.R230C (c.688C>T) and c.201-2A>G (near the canonical splice site). Other PPGL samples: Includes tumor samples from patients harboring pathogenic variants in Cluster 1B (*VHL*, n = 2) and Cluster 2 (*RET*, n = 5; *TMEM127*, n = 2). The *ACTB* gene was used as an endogenous control. (B) Western blot analysis of total SDHB protein levels in PPGL tumor samples, including both PHEO and PGL samples from the patient with CSS. Three SDHB-deficient tumor samples were used as controls. The specific gene variant for each sample is indicated in the figure. β-Tubulin protein was used as a loading control.

Our analysis revealed that *SDHB* transcript levels in the paraganglioma sample from the CSS patient were comparable to those observed in other PPGLs with genetic alterations in cluster 1B and cluster 2 genes (Fig. 2A). As expected, *SDHB* mRNA levels were reduced in a paraganglioma sample from a patient harboring the germline variant c.201-2A > G in *SDHB*, located near the canonical splice site and classified as likely pathogenic (Fig. 2A). This reduction is likely due to a second somatic event, leaving only the mutated allele. The resulting nonsense mutation appears to trigger mRNA degradation via the nonsense-mediated decay (NMD) pathway [17].

To further investigate *SDHB* expression, we performed Western blot analysis on tumor samples from the paraganglioma and pheochromocytoma of the patient with CSS. *SDHB* protein levels in these tumors were comparable to those observed in other PPGLs that are not deficient in *SDHB* expression (Fig. 2B). As a proof of concept, we analyzed 2 additional tumor samples with confirmed pathogenic *SDHB* variants: one carrying the missense variant c.688C>T (p.R230C) and another with the nonsense variant c.201-2A>G (Fig. 2B). As expected, *SDHB* protein levels were nearly undetectable in these *SDHB*-deficient tumors, consistent with the occurrence of a second somatic event leading to loss of heterozygosity (LOH) at the *SDHB* locus (Fig. 2B).

### Gene and Protein Expression of SLC25A11 in Tumor Samples

**Figure 3.**
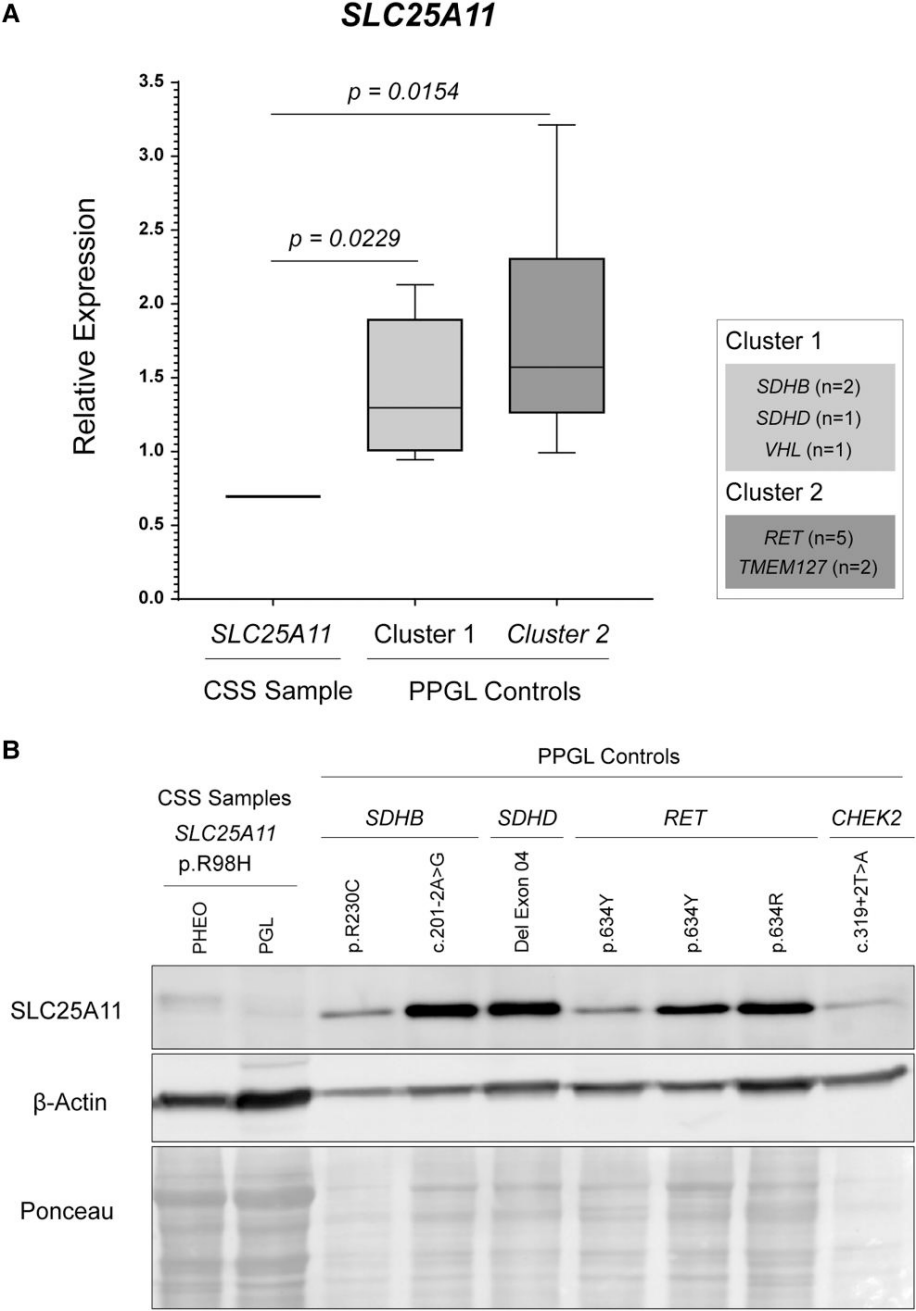
(A) Reduced mRNA levels of *SLC25A11* in tumor samples from the patient with Carney-Stratakis syndrome (CSS). Bars represent the mean ± SD from replicates. CSS sample (patient sample): Refers to the RNA obtained from the paraganglioma (PGL) of the CSS patient. Other samples: Includes pheochromocytoma (PHEO) and PGL from patients harboring pathogenic variants in Cluster 1 (*SDHB*, n = 2; *SDHD*, n = 1; *VHL*, n = 1) and Cluster 2 (*RET*, n = 5; *TMEM127*, n = 2). The *ACTB* gene was used as an endogenous control. (B) Western blot analysis of SLC25A11 protein levels in PHEO and PGL samples from the CSS patient. The gene variant for each sample is indicated in the figure. β-Actin was used as a loading control, and protein loading was further verified by Ponceau S staining.

Recent studies have identified germline pathogenic variants in the *SLC25A11* gene in patients with paragangliomas and metastatic disease [18]. These *SLC25A11* variants are frequently associated with LOH and a pseudohypoxia phenotype (cluster 1A), characterized by DNA hypermethylation, similar to tumors linked to *SDH*x genetic defects [18]. We then aimed to determine whether the germline variant of uncertain significance (VUS) c.293G>A in *SLC25A11* could be implicated in the development of neuroendocrine tumors in the patient with CSS. Strikingly, a marked reduction in *SLC25A11* expression was detected in tumor samples carrying the c.293G>A variant (0.69 ± 0.003) compared to cluster 1 (1.39 ± 0.45; *P* = .0229) and cluster 2 tumors (1.79 ± 0.71; *P* = .0154) (Fig. 3A). These findings suggest that the c.293G>A (p.R98H) variant exerts a deleterious effect on *SLC25A11* mRNA expression.

Consistent with the mRNA data, *SLC25A11* protein levels were markedly reduced in pheochromocytoma and paraganglioma samples carrying the germline *SLC25A11* c.293G>A variant compared to other tumors (Fig. 3B). This reduction is likely a result of impaired protein folding, leading to decreased stability and increased degradation.

### CNV Analysis at the *SLC25A11* Locus

We aimed to determine whether a second somatic event could explain the reduced levels of *SLC25A11* observed in the tumor samples from the patient with CSS. CNV analysis was performed on DNA from pheochromocytoma, paraganglioma, and GIST using an SNP array. A total of 27 825 SNPs from chromosome 17, where the *SLC25A11* gene is located, were analyzed. The SNP array revealed no CNV in chromosome 17 across all samples from the patient with CSS (Supplementary Fig. S2) [16]. Furthermore, PureCN analysis of WES confirmed these findings, as demonstrated by the results from the paraganglioma shown in Supplementary Fig. S3 [16]. Additionally, we used the Integrative Genomics Viewer (IGV) software to visualize the reads mapped to the *SLC25A11* locus, allowing us to investigate any coverage gaps that could indicate potential alterations in the whole-exome sequencing data from the germline and 3 tumor samples (Supplementary Fig. S4) [16]. However, no significant alterations were identified.

### Immunohistochemistry Reveals Evidence of Hypermethylation

**Figure 4.**
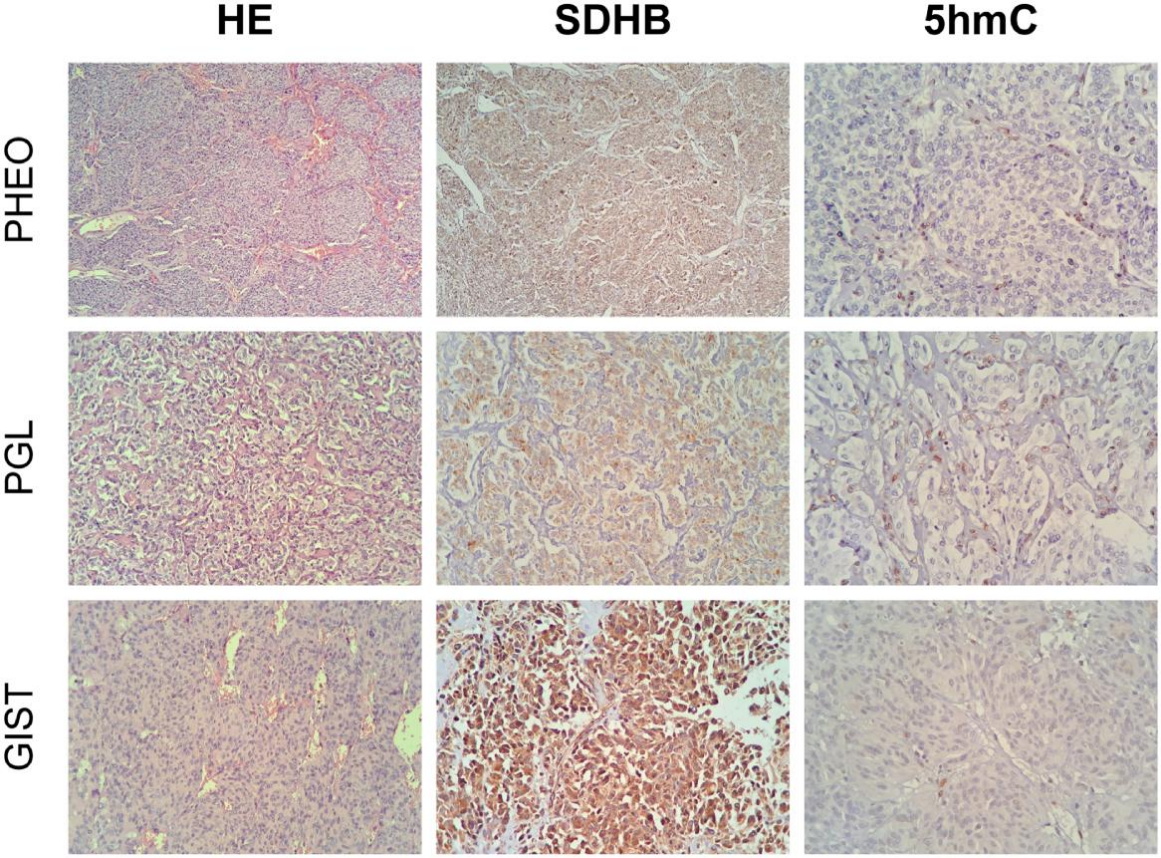
Immunohistochemical analysis of SDHB and 5-hydroxymethylcytosine (5hmC) in tumor samples from a patient with Carney-Stratakis syndrome, including pheochromocytoma (PHEO), paraganglioma (PGL), and gastrointestinal stromal tumors (GIST). The samples were stained with hematoxylin and eosin (HE), and images were captured at a magnification of 400×.

SDHB expression was analyzed in pheochromocytoma, paraganglioma, and GIST from the patient using immunohistochemistry. In addition to moderate to strong SDHB staining, we observed an absence of immunostaining for 5hmC in all 3 tumors, indicating a hypermethylated tumor profile (Fig. 4), which is characteristic of tumors associated with *SDHx* defects and paragangliomas in patients harboring pathogenic variants in *SLC25A11* [18]. The absence of 5hmC staining suggests increased levels of 5-methylcytosine, a marker of hypermethylation.

## Discussion

In this manuscript, we investigated the genetic etiology of a woman with CSS who presented with pheochromocytoma, para-aortic paraganglioma, and metastatic GIST. Genetic testing for pathogenic variants in the *SDHx* genes yielded negative results. Subsequently, we performed WES on paired blood and tumor samples (pheochromocytoma, paraganglioma, and GIST) to identify novel susceptibility genes for CSS. This analysis revealed a rare heterozygous germline variant (c.293G>A/p.Arg98His) in the *SLC25A11* gene, located at a highly conserved residue within the mitochondrial carrier domain. In silico analysis predicts this novel variant to be deleterious. Additionally, no somatic drivers were identified in the pheochromocytoma and paraganglioma, nor were any pathogenic variants in *KIT* or *PDGFRA* detected in the GIST.

The *SLC25A11* gene encodes the mitochondrial 2-oxoglutarate carrier (2-OGC), a crucial transporter that facilitates the exchange of 2-oxoglutarate (2-OG) and malate between the mitochondrial matrix and cytoplasm while maintaining electroneutrality. *SLC25A11* plays a central role in several metabolic pathways, including the malate-aspartate transport system, the oxoglutarate-isocitrate transport system, gluconeogenesis from lactate, and nitrogen metabolism [19]. The malate-aspartate transport system includes the 2-OGC (*SLC25A11*) and 2 aspartate-glutamate transporters, CITRIN (*SLC25A13*) and ARALAR (*SLC25A12*). This system plays a crucial role in regenerating the NADH pool in the mitochondrial matrix, enabling complex I to function properly [19]. Germline variants in the *SLC25A13* gene have been linked to hepatocellular carcinoma in Asian populations, indicating that the malate-aspartate transport system may play a role in the development of tumors [20]. Additionally, a gain-of-function variant in *GOT2*, the gene encoding mitochondrial aspartate aminotransferase, has been reported in a patient with paraganglioma, further supporting a link between malate-aspartate transport system dysfunction and PPGL [21].

A heterozygous missense variant in *SLC25A11* (c.715C>A/p.Pro239Thr) was first identified in a 46-year-old patient with a non-secreting abdominal paraganglioma, which exhibited a cluster 1A molecular profile in the absence of pathogenic variants in *SDHx* or *FH* [18]. Immunostaining for 5hmC and H3K9me3 further confirmed a hypermethylated phenotype. Somatic loss of the wild-type *SLC25A11* allele was observed by SNP array, which was consistent with the absence of protein staining. Germline *SLC25A11* variants were identified in 6 additional patients in a validation cohort of 639 PPGLs, 5 of whom had metastatic disease. LOH for *SLC25A11* was observed in 4 cases where tumor samples were available [18]. In vitro inactivation of *SLC25A11* induced both pseudohypoxic and hypermethylated phenotypes, leading to reduced levels of 2-OG, normal succinate levels, and elevated concentrations of aspartate and glutamate [18].

Rare PPGL patients with germline heterozygous *SLC25A11* variants have been reported, but no functional analyses were performed [22]. To further elucidate the pathogenicity of the germline heterozygous *SLC25A11* variant (c.293G>A/p.Arg98His) identified in our CCS patient, we demonstrated that *SLC25A11* expression was significantly reduced at both the mRNA and protein levels in the tumors of the CSS patient compared to PPGLs from patients with cluster 1 and 2 genetic defects. Additionally, all 3 tumors (pheochromocytoma, paraganglioma, and GIST) in our case exhibited a hypermethylation status similar to that of *SDH*-mutated tumors, characterized by a lack of 5-hmC immunostaining and retained SDHB protein expression.

Genetic alterations in *SLC25A11* have been associated with a higher risk of metastatic disease in 70% of the patients [18, 23]. In this report, a lymph node metastasis with positive staining for chromogranin and synaptophysin was identified in the pathological analysis. The patient did not experience recurrence of the PPGL but had an unfavorable outcome due to metastatic GIST. WES revealed 2 somatic *TP53* pathogenic variants in the GIST, which may have contributed to its aggressive behavior.

Buffet et al [18] demonstrated LOH for *SLC25A11* in all available PPGL samples from patients with germline *SLC25A11* variants, confirming its role as tumor suppressor gene. Here, we demonstrated a loss of *SLC25A11* tumor expression, but LOH was not detected by SNP array and pureCN analysis. Although LOH for *SLC25A11* was not observed in our study, WES of tumor samples did not reveal any somatic drivers in genes previously associated with PPGLs or GIST. Among the PPGLs without *SLC25A11* germline variants, *SLC25A11* expression was significantly lower in PPGLs from cluster 1 compared to those from cluster 2. Therefore, the hypermethylation phenotype of cluster 1 PPGLs reduces *SLC25A11* expression and might represent an epigenetic mechanism of LOH in the absence of CNVs.

CSS is an autosomal dominant disorder with incomplete penetrance caused by inactivating germline mutations in genes encoding the SDH subunits (*SDHB*, *SDHC*, and *SDHD*) [1]. Due to this genetic diversity, patients with CSS may exhibit a wide range of clinical presentations, which can contribute to its underdiagnosis [10]. It is important to note that the patient described here did not present with pulmonary chondroma, excluding the possibility of Carney triad. Additionally, the absence of pathogenic somatic variants in *KIT* and *PDGFRA* rules out the hypothesis of a sporadic GIST associated with multicentric PPGL.

In conclusion, a rare germline deleterious variant in *SLC25A11* was identified in a patient with CSS, who had a poor outcome due to metastatic disease. Moreover, the absence of somatic drivers, hypermethylation status, and loss of *SLC25A11* expression in the CSS tumors support *SLC25A11* as a new genetic etiology for CSS.

## Data Availability

The data supporting the findings of this study are available from the corresponding author upon reasonable request.

## Abbreviations

2-OG: 2-oxoglutarate
2-OGC: 2-oxoglutarate carrier
5hmC: 5-hydroxymethylcytosine
CNV: copy number variation
CSS: Carney-Stratakis syndrome
GIST: gastrointestinal stromal tumors
LOH: loss of heterozygosity
MLPA: multiplex ligation-dependent probe amplification
MIBG: meta-iodobenzylguanidine
MRI: magnetic resonance imaging
PPGL: pheochromocytoma and/or paraganglioma
SDH: succinate dehydrogenase
SNP: single-nucleotide polymorphism
WES: whole-exome sequencing
